# THE HISTORY OF BEAUTY AND THE AESTHETICS OF LONGEVITY

**DOI:** 10.1093/geroni/igz038.1126

**Published:** 2019-11-08

**Authors:** Naomi Woodspring

**Affiliations:** University of the West of England, Bristol, United Kingdom

## Abstract

A long standing cultural narrative is aging appearance is neither attractive nor acceptable. This has not always been the case; the aesthetics of older appearance has been appreciated other times in history. Significant numbers of older people in the public sphere, as a result of the longevity revolution, has created a sense of visibility of among older people, particularly women. The aim of this qualitative study was to explore current notions of beauty and age among the postwar generation. A diverse group of thirty-four women and men (born between 1945 -1955) from the US and the UK were interviewed with a focus on their own self-presentation and the acts of seeing and being seen. This paper explores the some of the findings from this study which asked the central question – can old people be beautiful and, if so, how is age and beauty defined? The majority of research participants answered in the affirmative and responded with clearly defined notions of age and beauty. The findings found significant gender differences; not within the central research question but in regard to their own appearance. There were also significant gender differences in terms of a ‘competitive’ or ‘cooperative’ gaze when observing other older people. This small study invites further research and points to a possible shift in the aesthetics of old age, in part, as a result of the longevity revolution. It provides an outline for further exploration of the importance of appearance, meaning, and a sense of self in old age.

